# Ascending aortic wall shear stress and distensibility are different in patients with corrected atrioventricular septal defect compared to healthy controls: a comprehensive CMR and 4D flow MRI evaluation

**DOI:** 10.1186/1532-429X-18-S1-P162

**Published:** 2016-01-27

**Authors:** Bernadette Elders, Pieter J van den Boogaard, Emmeline Calkoen, Nico A Blom, Albert de Roos, Jos J Westenberg, Arno Roest

**Affiliations:** 1 Pediatric Cardiology, LUMC, Leiden, Netherlands; 2 Radiology, LUMC, Leiden, Netherlands

## Background

In patients with an atrioventricular septal defect (AVSD), the left ventricular outflow tract (LVOT) and the ascending aorta (AAo) are located more anteriorly due to the position of the common AV valve. This may alter proximal aortic flow, and we hypothesize that this can result in impaired LV systolic function and AAo wall degeneration. We aimed to quantify differences in AAo wall shear stress (WSS), distensibility (Dist), aortic arch pulse wave velocity (PWV) and LV ejection fraction (EF) in these patients versus healthy controls using cardiovascular magnetic resonance (CMR) and four dimensional (4D) flow MRI.

## Methods

In 28 patients after AVSD correction (age 27 ± 13) and 28 healthy volunteers (age 25 ± 13, p = 0.52), whole-heart 4D flow MRI was performed on 3T MRI with free breathing, three-directional velocity encoding of 150 cm/s in all directions, spatial resolution 2.3 × 2.3 × 3.0-4.2 mm^3^ and 30 reconstructed phases. Additionally, high-temporal 2D phase-contrast MRI with through-plane velocity encoding transecting the thoracic aorta was performed to determine aortic arch PWV from transit-time propagation of the flow rate-time curve. Brachial cuff blood pressure was measured and AAo luminal area distention was measured at the sinotubular junction (STJ) to calculate AAo Dist. LVEF was obtained from multi-slice multi-phase cine short-axis planimetry using steady-state free-precession. WSS was calculated using CAAS MR 4D Flow 1v0 software (Pie Medical Imaging, Maastricht, The Netherlands) obtained at peak systole at STJ and averaged over the axial circumference. LVEF, Dist and PWV were calculated with in-house developed software. Both groups were compared using non-parametric t-tests.

## Results

Patients showed a higher mean AAo WSS versus controls (701 ± 327 vs 352 ± 125 mPa, p < 0.001). Peak WSS was increased in patients (1069 ± 430 vs 656 ± 216mPa, p < 0.001). In patients, decreased AAo Dist was observed compared to controls (5.2 ± 3.1 vs 9.2 ± 6.2 10^-3^ mmHg^-1^, p = 0.005). LVEF was significantly different between patients versus controls (55 ± 6% vs. 61 ± 5%, p < 0.001).No correlation was found between mean WSS and AAo Dist in patients (r = 0.06) or controls (r=-0.22) nor between mean WSS and PWV in patients (r=-0.12) or controls (r = 0.12). No significant difference in PWV was observed in patients versus controls (4.9 ± 1.3 vs 5.4 ± 1.3 m/s, p = 0.20).

## Conclusions

In patients with corrected AVSD, significantly higher WSS and decreased LVEF and Dist in the proximal ascending aorta was found using CMR and 4D flow MRI. Although no direct correlation between WSS and aortic Dist was found, our observations suggest that in these patients, ascending aortic flow is altered causing elevated WSS and possibly influencing local parameters of aortic function related to the more anterior positioned LVOT. Future research will provide more insight in the influence of anatomical deviation of the LVOT on the flow in the ascending aorta.

**Figure 1:**
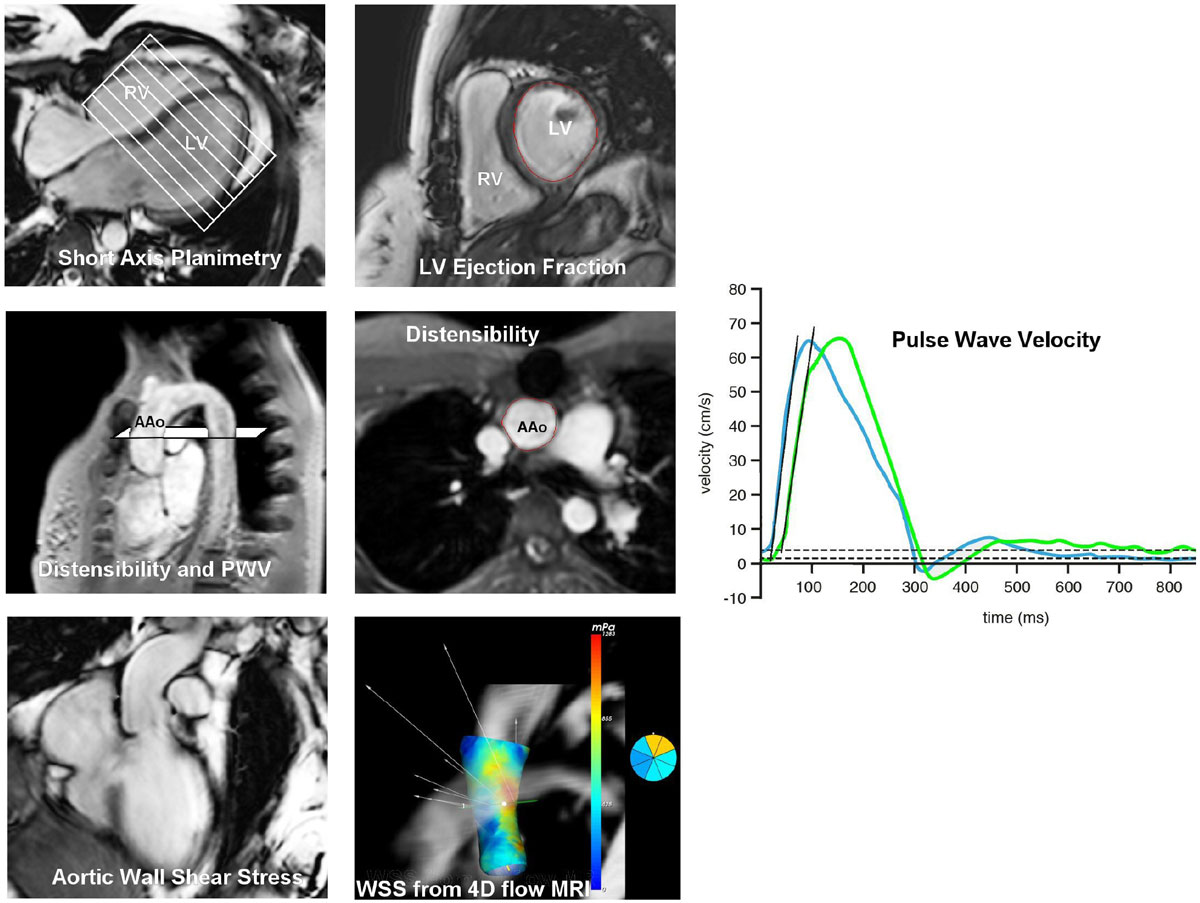
**CMR evaluation: LV short-axis evaluation to calculate LV ejection fraction (top row), time-resolved 2D velocity encoded MRI to determine Pulse Wave Velocity over the aortic arch and Distensibility of the ascending aorta (AAo) (middle row), 4D flow MRI to determine the wall shear stress in the ascending aorta (bottom row)**.

